# Comparison of prevalence, clinical evolution, and vaccination rate against covid 19 in a population of patients diagnosed with dual depression and non-dual depression

**DOI:** 10.1192/j.eurpsy.2022.1310

**Published:** 2022-09-01

**Authors:** S.L. Romero Guillena, A.M. Dopico Cervera, A. Pérez Romero

**Affiliations:** UGC Salud Mental Virgen Macarena, , Psychiatry, seville, Spain

**Keywords:** DUAL DEPRESSION, vaccination rate, Prevalence, covid 19

## Abstract

**Introduction:**

Since the beginning of the pandemic, 4,745,519 cases, 396,878 hospitalizations and 82,884 deaths with COVID-19 have been reported in Spain. As of August 24, 2021, 76.4% of Andalusians over 12 years of age have the complete vaccination regimen

**Objectives:**

Main: to calculate the prevalence of COVID 19 infection, clinical evolution and vaccination rate in a population of patients diagnosed with dual depression. Secondary: compare these data with those obtained in patients diagnosed with non-dual depression

**Methods:**

Retrospective descriptive study. The study population is made up of patients diagnosed with dual depression and non-dual depression (according to DSM 5 criterion). Infection, admission, death, and vaccination data were obtained from the patient’s medical history

**Results:**

Of the 10 patients diagnosed with dual depression, the prevalence of COVID 19 infection, since the beginning of the pandemic is 0% and of the 30 patients diagnosed with non-dual depression the prevalence is 3.33% (1/28). Of the patients with COVID 19 infection, none required hospital admission and no deaths occurred. The vaccination rate in the group of patients with dual depression is 30% (3/10) and in the group of non-dual depression is 86.66% (26/30), finding statistically significant differences (P<0.01) between both groups.

**Conclusions:**

In our study the prevalence of COVID 19 infection in patients diagnosed with dual depression is 0% and the vaccination rate is 30%. While in patients with non-dual depression the prevalence is 3.33%, there were no admissions, no deaths and the vaccination rate is 86.66%.

**Disclosure:**

No significant relationships.

